# Enrollment, retention, and strategies for including disadvantaged populations in randomized controlled trials: a systematic review protocol

**DOI:** 10.1186/s13643-021-01790-7

**Published:** 2021-08-18

**Authors:** Abigail LaPlante, Renata W. Yen, Talia Isaacs, Joanna Crocker, Zsofia Demjen, Danielle Schubbe, Alice M. Kennedy, Jaclyn Engel, Nancy O’Brien, Carla Richters, Marie-Anne Durand

**Affiliations:** 1 The Dartmouth Institute for Health Policy & Clinical Practice, Dartmouth College, Williamson Translational Research Building, Level 5, 1 Medical Center Drive, Lebanon, NH 03756 USA; 2 Tufts University School of Medicine, 145 Harrison Ave, Boston, MA 02111 USA; 3 UCL Centre for Applied Linguistics, UCL Institute of Education, University College London, 20 Bedford Way, London, WC1H 0AL UK; 4 Nuffield Department of Primary Care Health Sciences, University of Oxford, Radcliffe Primary Care Building, Woodstock Rd, Oxford, OX2 6GG UK; 5 Patient and Family Advocate, Dartmouth Hitchcock Medical Center Office of Patient Experience, 1 Medical Center Drive, Lebanon, NH 03756 USA; 6 UMR 1027, équipe EQUITY, Faculté de Médecine, 37 Allées Jules Guesde, 31000 Toulouse, France

**Keywords:** Disadvantaged populations, Trial recruitment, Randomized controlled trials, Enrollment, Retention, Health interventions, Health, Disparities

## Abstract

**Background:**

Many randomized controlled trials fail to reach their target sample size. When coupled with the omission and underrepresentation of disadvantaged groups in randomized controlled trials, many trials fail to obtain data that accurately represents the true diversity of their target population. Policies and practices have been implemented to increase representation of disadvantaged groups in many randomized controlled trials, with some trials specifically targeting such groups. To our knowledge, no systematic review has quantified the enrollment metrics and effectiveness of inclusion and retention strategies in randomized controlled trials focused on disadvantaged populations specifically.

**Methods:**

We will conduct a systematic search across EMBASE, MEDLINE, Web of Science, and CINAHL as well as grey literature, conference proceedings, research monographs, and Google Scholar from inception onwards. We will include randomized controlled trials where at least 50% of enrolled participants are considered to be disadvantaged, as per the RCT authors’ definition and in line with our inclusion criteria. Two independent researchers per article will conduct preliminary title and abstract screening, subsequent full text review, and data extraction for the selected trials, with a third reviewer available to resolve conflicts. We will assess the quality of all included studies using specific criteria regarding data reporting, external validity, and internal validity. We will combine all selected studies and conduct a narrative synthesis to assess enrollment metrics. If there is sufficient homogeneity and sufficient trials comparing recruitment strategies within disadvantaged populations, we will conduct a random effects meta-analysis to evaluate the effectiveness of strategies designed to maximize the inclusion of disadvantaged populations in randomized controlled trials.

**Discussion:**

The findings of this systematic review will establish baseline recruitment and enrollment metrics of trials targeting disadvantaged populations to elucidate the scope of the challenge of recruiting such populations. We hope that our findings will promote future research on the distinct barriers that may prevent disadvantaged populations from participating in health intervention research, will encourage more trials exploring effective, tailored recruitment strategies, and will establish a foundation to track future progress in the recruitment of disadvantaged populations.

**Trial registrations:**

PROSPERO ID: CRD42020152814

**Supplementary Information:**

The online version contains supplementary material available at 10.1186/s13643-021-01790-7.

## Background

Members of disadvantaged populations, such as those disadvantaged by virtue of socioeconomic status, race/ethnicity, gender, and/or education level, tend to be underrepresented in health and medical research [1, 2]. Failing to recruit a sample of research participants who represent the diversity of the target population threatens the generalizability of the trial findings, as inferences made about the safety and efficacy/effectiveness of the interventions being tested for these groups may prove to be incorrect [3]. The generalizability of the findings is instead limited to those sufficiently similar to the study population, often excluding those with the greatest burden of health issues: disadvantaged populations [2, 4, 5]. In order to address these inequities, some trials specifically target disadvantaged populations, aiming to recruit and enroll research participants from population subgroups [3, 4, 6–10].

Despite the targeted inclusion of disadvantaged groups in some randomized controlled trials (RCTs), there is a paucity of comprehensive data on participation. Previous reviews have focused on only one facet of the recruitment, enrollment, and retention triad, rather than exploring the nuances of each. While the terms recruitment and enrollment are often used interchangeably, they are distinct concepts; recruitment is defined as the proportion of people who enrolled, out of all people assessed for eligibility while enrollment is defined as the proportion of people who enrolled out of all people determined to be eligible [11, 12]. Still other reviews tend to focus on certain conditions (i.e., cancer, obesity, heart disease) rather than disadvantaged populations in trials more generally, irrespective of health condition or setting [3, 4, 6, 7].

Additionally, the existing literature is terminologically complex [13]. RCTs and trials methodology literature have referred to the recruitment and enrollment of other or sidelined populations as “underrepresented,” “hidden,” “understudied,” “hard-to-reach,” “underserved,” “vulnerable,” and/or “disadvantaged,” and this list is non-exhaustive [2, 3, 12, 14–19]. The first four labels arguably frame the inequity of research participation primarily in research-centric terms. Studies may elaborate by directly emphasizing that the inclusion of such target groups can be challenging to access from researchers’ perspective and/or that their inclusion could enhance external validity. Other studies characterize research participation primarily from a person-centered, social justice, and health equity lens, as implied by the last three listed labels. Regardless of the term(s) that the authors choose, studies may, of course, emphasize both research and social facets of the inclusion of such groups.

In the context of this study, we operationalize one commonly used term, the construct of “disadvantage,” in relation to RCTs. We define disadvantage as social, cultural, or financial disparities that imply environmental, historical-structural, or social restriction to opportunities for health [14, 15, 19–23]. We recognize that disadvantage may involve one or more specific attributes, contexts, or group types. We will utilize the PROGRESS-Plus framework to guide our review. PROGRESS-Plus is a health equity framework intended to ensure that social determinants of health are considered when conducting research; the acronym stands for place of residence, race/ethnicity/culture/language, occupation, gender/sex, religion, education, socioeconomic status, and social capital [23].

In the general population, it is estimated that 50–56% of RCTs fail to reach their target enrollment [24–26]. It remains unclear how the barriers specifically faced by disadvantaged populations influence enrollment rates. No review or meta-analysis currently exists that systematically evaluates participation rates and recruitment strategies in RCTs targeting disadvantaged populations. In order to address these gaps, the primary aim of our systematic review is to assess (1) recruitment rate (defined as the proportion of people who enrolled, out of all people assessed for eligibility); (2) enrollment rate (defined as the proportion of people who enrolled out of all people determined to be eligible); (3) enrollment yield (defined as the proportion of enrolled participants compared to initial target sample size); and (4) retention rate (defined as the proportion of people who enroll and who complete the study) of disadvantaged populations included in RCTs targeting disadvantaged populations [11]. Our secondary aim is to assess the effectiveness of strategies designed to maximize the enrollment and retention of disadvantaged populations in RCTs.

## Methods

### Information sources and search strategy

The present protocol has been registered with the PROSPERO database (registration number CRD42020152814) and is being reported in accordance with the reporting guidance provided in the Preferred Reporting Items for Systematic Reviews and Meta-Analyses Protocols (PRISMA-P) statement [27] (see checklist in Additional file 1).

We developed the search strategy with research and education librarians at Dartmouth College Biomedical Libraries and at the University College London (UCL) Institute of Education, two major research-intensive universities in the USA and UK. We piloted the search strategy in Ovid MEDLINE. We will perform electronic searches in EMBASE, MEDLINE, Web of Science, and CINAHL from inception onwards (see draft search strategy in Additional file 2).

We will conduct the search using keywords written in English using English language databases. We will include peer-reviewed journal articles, grey literature, conference proceedings, and research monographs written in English, German, Hungarian, and French according to reviewer language skills. We will exclude book chapters, conference abstracts, and protocol papers.

Two independent reviewers will manually search the reference list of each included primary and relevant review article to identify studies that have not been picked up in the electronic search. We will also perform a citation search using the ‘cited by’ option in Google Scholar for each included primary article. We will use key themes to search Google Scholar for RCTs that meet our inclusion criteria that were not picked up in our main database searches. Two reviewers will manually search the first 100 hits in Google Scholar while documenting any discrepancies in the search results.

We will search grey literature (i.e., technical reports, works in progress). We will search ClinicalTrials.gov and the World Health Organization International Clinical Trials Registry Platform (WHO ICTRP) for RCTs that meet our inclusion criteria that were not picked up in our main database searches. We will similarly search ORRCA (Online Resource for Recruitment research in Clinical triAls) by adapting the search strategy to examine relevant categories under “trial conduct” and “recruitment information.”

### Screening and study selection

We conducted a preliminary search in Google Scholar and ORRCA to identify existing systematic reviews examining disadvantaged populations and to assess the volume of potentially included articles [28]. We assessed literature reviews and the most cited RCTs from both searches.

We will review and consider all search results for inclusion using Rayyan, a freely available web application designed for screening systematic review records [29]. Two researchers per article will independently assess the title and abstract of each retrieved record and the full-text articles meeting the inclusion criteria. We will resolve any disagreements on inclusion by arbitration with a third reviewer.

### Eligibility criteria

#### Types of study designs

We will include all RCTs where the RCT authors explicitly state that they targeted the inclusion of disadvantaged populations. The RCT must be conducted in a healthcare setting. This can include lay care (a health worker who is trained to deliver healthcare but who has not received a formal professional certificate or degree), primary care, secondary care, community centers, telehealth, etc. [30].

We define RCTs as any research study that prospectively and randomly assigns individuals or groups of humans to either a health-related intervention(s) or to a control group [31, 32]. This excludes randomized feasibility trials and randomized pilot trials. Interventions could include but are not restricted to drugs, cells, and other biological products, surgical procedures, radiological procedures, devices, behavioral treatments, process of care changes, preventive care, and educational interventions.

#### Types of participants

RCTs will be included in the initial title and abstract screening if the RCT authors identify their participants as a disadvantaged group concordant with one or more of the following PROGRESS-Plus criteria: place of residence, race/ethnicity/culture/language, occupation, gender/sex, religion, education, socioeconomic status, and social capital [14, 15, 19–23]. Due to the terminologic complexity regarding disadvantaged status, author framing of disadvantage using terms such as “vulnerable,” “hard-to-reach,” or “underserved” will merit inclusion.

During the full-text review, RCTs will be assessed further to ensure that the author frames the population as disadvantaged by linking the population’s PROGRESS-Plus characteristics with differential opportunities for health (see Table 1).

**Table 1:** Examples of Author framing of disadvantage from preliminary search

Article	PROGRESS-Plus category	Author’s framing of disadvantage
Apter et al. [33]	Socioeconomic status	“Asthma disproportionately affects low-income and minority adults, particularly African and Puerto Rican Americans.”
Breitkopf et al. [34]	Race/ethnicity/culture/language	“Approaches that target low-income and minority women are especially important, as socioeconomically disadvantaged and minority women bear a disproportionate burden of cervical cancer morbidity and mortality.”
Zoellner et al. [35]	Place of residence	“The Talking Health trial was developed to address these gaps in the literature and to target needs of the medically-underserved Appalachian region of rural southwest Virginia... There are also notable socioeconomic (median income, percent population below poverty, educational achievement, etc.) and literacy proficiency disparities within this region, as compared to state and national averages.”

RCTs will be included if at least 50% of enrolled participants were from disadvantaged groups, according to the above operational definition [36].

We will include RCTs in which the participants receiving the intervention are patients, health professionals, or members of the general public from disadvantaged groups, as defined by the author. We will include trials that target adults (age 18 or older) with or without an illness

#### Types of outcome measures

In order to prevent duplication of included RCTs and included participants, we will only include articles that report on the primary outcome(s) of the included RCTs. We will include RCTs in which the *primary outcome measures* are health related, including affective-cognitive, behavioral, and/or physiological outcomes. We will not include RCTs where the primary outcome was recruitment rate, enrollment rate, or retention rate in order to isolate the analysis to medical outcomes.

### Assessment of methodological quality

We will rate the methodological quality of included RCTs using selected and recruitment-focused criteria on data reporting, internal validity, external validity, specifically adapted from Black and Down’s checklist for the assessment of the methodological quality (items 3, 9, 12, and 21—see Table 2) [37].

**Table 2:** Quality assessment measures

Checklist item	Assessment measure
3	Are the characteristics of the patients included in the study clearly described?
9	Have the characteristics of patients lost to follow-up been described?
12	Were those subjects who were prepared to participate representative of the entire population from which they were recruited?
21	Were the patients in different intervention groups (trials and cohort studies) or were the cases and controls (case-control studies) recruited from the same population?

Two independent assessors will use the checklist for all included studies. An answer of “yes” to any of the measures correlates with a score of 1, while an answer of “no” or “unable to determine” correlates with a score of 0. Thus, the total quality assessment score for each article can range from 0 to 4. Each assessor will be trained on using the checklist before initiating the quality assessment. We will resolve any discrepancies by discussion and consensus.

### Data extraction

We will perform independent double data extraction, using a pre-designed form, adapted from the Cochrane Effective Practice and Organization of Care (EPOC) collection checklist [38]. Two researchers will pilot the data extraction form independently using three studies purposively selected for this pilot exercise. We will resolve inconsistencies by discussion.

We will extract information about (1) the author(s); (2) publication year; (3) country(ies) in which data collection took place; (4) study design (RCT, cluster RCT, pilot RCT, etc.); (5) condition(s) targeted; (6) type(s) of intervention(s); (7) research aim(s) and questions; (8) participant characteristics as they do/do not relate to disadvantaged status and sample size; (9) author’s framing of participants’ disadvantaged status as it relates to differential opportunities for health (10) setting (lay care, primary care, secondary care, community centers, telehealth, etc.); (11) recruitment strategies and comparator (if applicable); (12) target enrollment rate; (13) recruitment rate (by strategy, if applicable); (14) enrollment rate (by strategy, if applicable); (15) retention rate (by strategy, if applicable); and (16) outcome measures (primary and secondary).

### Data synthesis

We will synthesize primary studies and produce a narrative review to descriptively assess for heterogeneity. The data from each RCT, including study characteristics, context, participant characteristics, exposures, and enrollment and recruitment metrics, will be used to build evidence tables according to the Synthesis without meta-analysis (SWiM) reporting guidelines [39]. Using the evidence tables, we will qualitatively summarize the effect of recruitment strategies across disadvantaged populations on recruitment metrics.

We will also assess heterogeneity using the chi-square test and *I*^2^ test [40]. If there is sufficient homogeneity (*p* > .10 and *I*^2^ < 50%) and sufficient trials reporting recruitment rate, enrollment rate, enrollment yield, and retention rate by disadvantaged status, we will pool studies into a meta-analysis to assess the effectiveness of strategies designed to maximize the inclusion of disadvantaged populations in RCTs. This is a dichotomous outcome that would be assessed via relative risk to assess for differences in outcome by disadvantaged status. We would calculate standardized mean differences if any important continuous outcomes arise. We will use a random effects model in our analysis to account for the variability in included disadvantaged populations and differences between subpopulations.

If the data are not appropriate for a meta-analysis, we will summarize these outcomes using SWiM reporting guidelines [39]. We will generate summary measures of our outcomes of interest across the studies to understand the distribution of recruitment, enrollment, and attrition rates across the included studies, and we will compare these results with established published rates of recruitment, enrollment, and attrition in the general population. We will also conduct subgroup analyses to look at these rates by recruitment approach and retention strategies.

We will examine how missing data is biasing our results; we will use funnel plots and Egger’s regression test to evaluate potential publication bias. We will look at the overall sample size of the study and see if we are missing any studies—e.g., studies with lower sample sizes and large attrition. Statistical significance will be assumed at *p* < .05.

### Patient and public involvement

We will have ongoing participation of a patient partner throughout the duration of this project. The partner is a community member with lived experience with one or more of the social barriers described above. They showed an interest in contributing to research on the inclusion of disadvantaged participants in RCTs and have contributed to the development of the protocol. They will provide future guidance on the final systematic review as well as the communication and messaging of the review results.

## Discussion

The findings of the systematic review will enhance the data on disadvantaged population participation in RCTs, by compiling recruitment data for this population as well as by identifying evidence-based strategies for increased enrollment and retention. The baseline recruitment and enrollment metrics established in this systematic review will elucidate the scope of the challenge of recruiting such populations, especially when contextualized in the existing literature on recruitment metrics in the general population. The COVID-19 pandemic has made clear that such information is critical; disadvantaged populations are disproportionately burdened by the disease yet difficult to recruit for ongoing vaccine trials [41].

We hope that our findings will promote future research on the distinct barriers that may prevent disadvantaged populations from participating in RCTs, will encourage more trials exploring effective, tailored recruitment strategies, and will establish a foundation to track future progress in recruiting disadvantaged populations.

### Limitations

This systematic review may be limited in that not all studies will adhere to a standard reporting guideline, and thus will not report all aspects of the recruitment and enrollment triad. Further, we anticipate that authors will employ different criteria and language for disadvantage within the PROGRESS-Plus framework; however, we accept this knowing that disadvantage is indeed context dependent. We anticipate this to be a limitation in our systematic review, as it may result in substantial heterogeneity for most outcomes.

### Dissemination and amendments

Results will be disseminated through conference presentations and publication in a peer-reviewed journal. Any amendments made to this protocol when conducting the review will be outlined in PROSPERO and reported in the final manuscript.

## Supplementary Information

**Additional file 1.** PRISMA-P Checklist.

**Additional file 2.** Search Strategy.

## Data Availability

Data sharing is not applicable to this article as no datasets were generated or analyzed during the current study. Search strategy for MEDLINE, CINAHL, Web of Science, and EMBASE is included in Additional file 1.

## Abbreviations

RCTs: Randomized controlled trials
