# Supplementary material for: Enrollment, retention, and strategies for including disadvantaged populations in randomized controlled trials: a systematic review protocol
Source: Syst Rev. 2021 Aug 18;10:233. doi: 10.1186/s13643-021-01790-7 (PMC8372683; doi:10.1186/s13643-021-01790-7)
Supplement: Supplementary file 2 — Additional file 2. Search Strategy. [file 13643_2021_1790_MOESM2_ESM.docx]

Additional file 2: Search Strategy

**MEDLINE (via Ovid)**

1. Healthcare Disparities/ or Health Status Disparities/ or Social Determinants of Health/ or

((health or healthcare) adj inequalit*).ab. or ((health or healthcare) adj disparit*).ab. or

disadvantag*.ab. or exp Socioeconomic Factors/ or Working Poor/ or Poverty/ or exp

Social Class/ or socioeconomic status.ab. or exp Minority Groups/ or exp Race Factors/

or minority group*.ab. or ethnic group*.ab. or Gender Identity/ or Transgender Persons/

or Transsexualism/ or Bisexuality/ or exp Homosexuality/ or gender ident*.ab. or

transgender*.ab. or transexual*.ab. or bisexual*.ab. or homosexual*.ab. or lgbt*.ab. or

exp Disabled Persons/ or Developmental Disabilities/ or Intellectual Disability/ or

Learning Disabilities/ or disabled.ab. or developmental disabilit*.ab. or intellectual

disabilit*.ab. or learning disabilit*.ab. or physical disabilit*.ab. or Educational Status/ or

educational attainment.ab. or exp Information Literacy/ or Language Tests/ or exp Health

Literacy/ or low* health literacy.ab. or low* literacy.ab. or Rural Health/ or geographical

disadvantage.ab. or neighborhood disadvantage.ab. or geographic remoteness.ab. or

((poor or limit* or reduc*) adj7 health care access).ab. or Medically Uninsured/ or

Medical Indigency/ or underinsured.ab. or uninsured.ab. or numeracy.ab. or

Communication Barriers/ or language barrier*.ab. or Homeless Persons/ or no fixed

abode.ab. or homeless.ab. or migrant*.ab. or undocumented immigrant*.ab. or

transient.ab.

2. (Health* or Medic*).mp.

3. (enrol*ment or retention or recruitment or consent or agreed to or joined or stayed).ab.

4. 1 AND 2 AND 3

5. limit 4 to (clinical trial, all or randomized controlled trial)

6. limit 5 to “all adult (19 plus years)”^1^

1. While our systematic review age inclusion criteria is adults defined as 18 and older, due filter settings of this database, we had to search for “all adult (19 plus years)”
